# Healthcare-seeking behavior among pregnant women in the Chinese hierarchical medical system: a cross-sectional study

**DOI:** 10.1186/s12939-019-1037-8

**Published:** 2019-08-19

**Authors:** Guihao Liu, Yunlian Xue, Zhenzhu Qian, Liuna Yang, Yunbin Yang, Qingshan Geng, Xin Wang

**Affiliations:** 1 Guangdong Provincial People’s Hospital, Guangdong Academy of Medical Sciences, 106 Zhongshan II Yuexiu District, Guangzhou, Guangdong China; 2 Southern Medical University, 1023-1063 Shatai south road, Guangzhou, Guangdong China; 3 College of Humanities and Social Sciences, China Medical University, 77 Puhe Road, Shenbei New District, Shenyang, Liaoning China; 4 Guangdong Medical University, Songshan lake science and technology industrial park, Dongguan, Guangdong China

**Keywords:** Hierarchical medical system, Pregnant women, Environmental factors, Demographic factors, Antenatal care

## Abstract

**Background:**

Hierarchical medical systems are common in developed countries, but it’s not optimistic in China. This study aimed to identify the factors affecting healthcare-seeking behavior among pregnant women in Guangdong, China.

**Methods:**

We conducted a cross-sectional, observational survey, developed using the Andersen’s behavioral model. Pregnant women were randomly selected using a two-stage, stratified, random sampling method from hospitals in Guangdong, China. A multinomial logistic regression was used to identify statistically significant variables from aspect of environmental, demographic and pregnancy characteristics associated with pregnant women seeking healthcare at primary, secondary or tertiary hospitals.

**Results:**

A total of 1393 pregnant women returned the survey after attending 1 of 12 hospitals within 4 cities of the Guangdong province: 537 (38.5%) of the respondents attended a primary hospital, 437 (31.4%) a secondary hospital, and 419 (30.1%) a tertiary hospital. Women attending primary hospitals were more likely to live closer to the hospital, live rurally, and be educated to a lower level. Several factors were significantly associated with attendance at a secondary vs a primary hospital: the woman’s perceived necessity to seek maternal healthcare (OR 1.73, 95% CI [1.1,2.74]), the woman’s choice of delivery hospital (OR 1.45, 95% CI [1.01,2.07]), or urban living (OR 1.39, 95% CI [1.03,1.88]). Characteristics associated with attendance at a tertiary vs a primary hospital were: a history of pregnancy complications (OR 2.35, 95% CI [1.43,3.86]), travel to the hospital by public transport/taxi (OR 2.09/2.67, 95% CI [1.35,3.22]/ [1.45,4.92]), urban living (OR 1.58, 95% CI [1.14,2.18]), or a planned current pregnancy (OR 1.53, 95% CI [1.07,2.19]).

**Conclusion:**

Medical needs and convenience both play a role in the choice of hospital for antenatal care, and impact on equity utilization of health services. Pregnant women without risk factors and with higher levels of education should be a target population for guiding to choose a more proper level of hospital.

## Background

The maternal mortality rate is widely used to assess the quality of obstetric care in different countries and regions [1]. Over the last decade, the maternal mortality rate has dropped significantly in China, from 80 per hundred thousand in 1991, to 21.7 per hundred thousand in 2014 [2]. Comprehensive maternal care is vital in assuring the safety, and well-being women and their newborn children [3]. However, the under-utilized maternal healthcare services in China have affected the equity in utilization of health services. Seeking healthcare advice as a preventative method is the ideal way to reduce the risks of pregnancy for both the mother and unborn child. This type of healthcare service is generally high throughput in nature and attracts minimal investment, despite its great importance in promoting maternal and child health [4].

Healthcare-seeking behaviour can be understood as a person’s process of engaging (or not) with a particular health service. This includes aspects such as how symptoms are perceived and acted upon and how, as well as which type of and when, healthcare services are accessed [5]. Healthcare-seeking behavior has emerged as a tool to tackle perceived ill health by taking remedial actions [6]. A woman’s decision to seek healthcare is not an isolated event; rather, it is a composite result of her personal needs, social forces, actions of healthcare providers, and the location of services [7]. Andersen’s health behavior model systematically explores the factors which influence health service utilization [8]. The model includes four main parts: environmental factors, population characteristics, health behaviors, and health outcomes [9]. At its core, the model suggests that a person’s individual characteristics and healthcare needs influence their motivation and ability to access healthcare services; therefore, overall health service utilization is affected by population characteristics and their environment [10, 11]. Andersen’s model has been widely used in many fields, but particularly by healthcare decision makers, who use it to optimize health service utilization [12].

Chinese Hierarchical Medical System contains three-level medical institutions, primary, secondary and tertiary medical institutions. Compared with primary and secondary medical institutions, tertiary medical institutions have more experienced experts and advanced equipments. As a result, the actual demands may be ignored and patients tends to see doctors in the tertiary medical institution. This could aggravate the inequity of health seeking. Pregnant women usually have insufficient knowledge of pregnant symptoms [13]. It has been indicated that the equity of maternal and child health service utilization in China was improved from 2010 to 2015; however, economically developed areas and eastern areas were relatively concentrated on in this study [14]. Guangdong Province is in southern east of China and one of the most population provinces. After the promulgation of the comprehensive “two-child” policy, the number of pregnant women has increased markedly, which leads to a bigger challenge for equity of healthcare-seeking. Our previous studies indicated that the passing rate (71.4%) of healthcare seeking behavior of puerpera in Guangdong Province was low, and many factors influenced the healthcare-seeking behavior [15].

Although, some large studies have evaluated the environmental and demographic factors affecting healthcare-seeking behavior of pregnant women in developed countries [16–18], only a few studies in China have been found [19]. Due to the great demand of health service providing of gynaecology and obstetrics especially after the promulgation of the comprehensive “two-child” policy, it is important to promote an equitable and reasonable healthcare-seeking environment for pregnant women. This study aimed to discover the factors influencing healthcare-seeking behavior of pregnant women in Guangdong hierarchical healthcare system and put forward feasible measures to improve the equity of healthcare-seeking behavior of pregnant women.

## Methods

### Study setting and sampling

A cross-sectional survey conducted in hospitals around of Guangdong province in China. The study protocol was approved by the Guangdong Provincial People’s Hospital. Institutional Review Board and followed principle expressed in the Declaration of Helsinki. Pregnant women were recruited in a two-stage stratified sampling process. In the first stage, Guangzhou (middle area, 13.50 million population), Foshan (middle area, 7.43 million population), Shaoguan (north area, 2.93 million population) and Zhanjiang (west area, 7.24 million population) were selected. In terms of the aspect of areas division, Guangzhou and Foshan are in the middle area, Shaoguan is in the north area, Zhanjiang is in the west area. With regards to the population scale in 2015, the populations are 13.50 million, 7.43 million, 7.24 million and 2.93 million in Guangzhou, Foshan, Zhanjiang and Shaoguan, respectively [20]. In addtion, based on the GDP ranking in 2015, of all the 21 cities, Guangzhou is No.1, Foshan is No.3, Zhanjiang is No.7, Shaoguan is No.16 [21]. The comprehensive consideration of area, population scale and GDP ranking indicate that these chosen cities are sufficiently represented the socioeconomic backgrounds of Guangdong Province. In the second stage, target hospitals were sampled from the selected cities, stratified by the level of specialized care available. In each city, one general hospital or maternal and child care service center was chosen from the primary, secondary, or tertiary medical institutions respectively. In total, 12 hospitals were chosen, and pregnant women who sought for healthcare in a particular time period in these hospitals were investigated.

### Respondents

Respondents were women who had antenatal care with gestational week more than 28 at one of the selected hospitals, and get into the investigation during Apr. 2017 and Oct. 2017. Written informed consent was obtained from each respondent prior to completion of the survey. We planned to survey approximately 1500 pregnant women, equally distributed among the 12 sampled hospitals.

### Survey

Surveys were developed using the Andersen’s behavioral model and distributed to respondents by the doctors providing antenatal care. The survey contained 25 questions, which were selected by 24 experts in obstetrics and gynecology, epidemiology and health statistics, psychology, and health policy during two rounds of Delphi consultation. The details of establishment of comprehensive evaluation index system of maternal healthcare-seeking behavior were described in our previous study [22]. The questions formed two broad categories: 1) environmental factors that impact healthcare-seeking behaviors: urban vs rural living, annual per capita household income, knowledge of the national maternal healthcare policies, previous participation in either health education or consulting, a medical examination prior to pregnancy, a health assessment/medical guidance, or a network-based health education program; 2) population characteristics that impact healthcare-seeking behaviors: age, highest level of education, marital status, number of previous pregnancies, number of previous abortions, number of prior live births, history of pregnancy complications, whether the current pregnancy was planned, opinion on the necessity for maternal healthcare, feelings on the necessity for an antenatal examination when feeling good, whether it was in the women’s power to decide on the location of delivery, health insurance schemes, distance from the hospital, mode of transport to the hospital, waiting time to see the doctor, subjective feelings about maternal healthcare, feelings on how the antenatal advice applies to their personal situation, or if personal privacy was respected during antenatal care. These questions were all contained in the single factor analysis, and only the ones had statistically significant difference were brought into multiple factors analysis.

### Data processing and analyses

Surveys were completed between Apr. 2017 and Oct. 2017. Prior to the start of the study in each hospital, the local team received training on survey and data collection techniques from the research team. Questions could be misunderstood were detailed explained to respondents. Data were checked for completeness and inconsistencies, coded, and entered into Epidata 3.0. Descriptive analysis was used for the demographic characteristics of the participants. A chi-square test was used to identify variables associated with healthcare seeking at each level of hospital. Those variables with statistical significance were included in a multinomial logistic regression analysis (test of parallel lines of ordinal logistic analysis was rejected). Two-sided *p*-values < 0.05 were considered to indicate statistical significance. All statistical analysis was done using SPSS software, version 20.0.

## Results

In total, 1420 of the 1500 distributed survey were recollected. Among these, 27 were considered invalid due to incomplete data. The valid returned rate was 92.9%.

### Demographic characteristics of the respondents

Overall, 537 (38.5%) of the respondents attended a primary hospital, 437 (31.4%) a secondary hospital, and 419 (30.1%) a tertiary hospital. The 1393 pregnant women aged from 17 to 43 years old, with a mean (±standard deviation) of 28.0 ± 4.8 years. Only 100 respondents were aged over 35 years (7.2%). About half (647 [46.4%]) of the respondents had a college degree or above, while more than half (769, 55.2%) resided in a rural area of the province. The median distance to the hospital was 5 km, but 20.6% (287) of the respondents travelled more than 10 km. In addition, 1219 (87.5%) respondents’ subjective feelings about maternal healthcare were good, 1353 (97.1%) respondents’ subjective feeling about personal privacy were respected during antenatal care.

Among the Respondents, 725 (52.0%) reported annual per capita household income of less than 50,000¥. The majority (1207 [86.6%]) of respondents were covered by health insurance schemes, of which medicare accounted for 56.3% (679), new rural cooperative medical system accounted for 32.3% (390), free medicare accounted for 9.7% (117), and commercial insurance accounted for 1.7% (21). The median of waiting time to see the doctor was 20 min; however, 20.2% (281) of the respondents waited less than 15 min, and 18.6% (259) more than 30 min. Notably, we found that the incidence of abortion and pregnancy complications among women aged over 35 years old were 29.9 and 17.0%, higher than in younger women, respectively.

### Factors associated with healthcare-seeking at primary, secondary or tertiary hospitals

Univariate chi-square test identified 14 demographic variables that were significantly different between the hospital levels. Pairwise comparisons were also done (Table 1).

**Table 1:** Comparison of factors associated with pregnant women attending different primary, secondary and tertiary hospitals (*n* = 1393)

Variable, n (%)	Hospital level			*P-value*
	Primary	Secondary	Tertiary	
Environmental factors				
Urban vs rural living				
Urban	185 (34.5)	216 (49.4)	223 (53.2)	< 0.001
Rural	352 (65.5)	221 (50.6)	196 (46.8)	
Annual per capita household income				
< 50,000¥	317 (59)	245 (56.8)	163 (39.1)	< 0.001
≥ 50,000¥	220 (41.0)	186 (43.2)	254 (60.9)	
Aware of the national maternal healthcare policies?				
Yes	339 (63.1)	296 (67.7)	258 (61.6)	0.143
No	198 (36.9)	141 (32.3)	161 (38.4)	
Previously participated in any health education or consulting before pregnancy?				
Yes	282 (52.5)	206 (47.1)	216 (51.7)	0.215
No	255 (47.5)	231 (52.9)	202 (48.3)	
Previously participated in medical examination before pregnancy?				
Yes	304 (56.6)	280 (64.1)	266 (63.6)	0.026
No	233 (43.4)	157 (35.9)	152 (36.4)	
Previously sought a health assessment or medical guidance before pregnancy?				
Yes	193 (35.9)	125 (28.6)	134 (32.1)	0.051
No	344 (64.1)	312 (71.4)	284 (67.9)	
Previously participated in network-based health education?				
Yes	109 (20.3)	93 (21.3)	73 (17.5)	0.345
No	428 (79.7)	344 (78.7)	345 (82.5)	
Demographic and pregnancy characteristics				
Age, years				
< 35	514 (95.7)	412 (94.3)	367 (87.6)	< 0.001
≥ 35	23 (4.3)	25 (5.7)	52 (12.4)	
Highest education level				
Junior high school or below	160 (29.8)	86 (19.7)	70 (16.7)	< 0.001
High school	206 (38.4)	117 (26.8)	107 (25.5)	
College or above	171 (31.8)	234 (53.5)	242 (57.8)	
Number of previous pregnancies				
1 time	309 (57.5)	281 (64.3)	223 (53.2)	< 0.001
2 times	132 (24.6)	123 (28.1)	114 (27.2)	
≥ 3 times	96 (17.9)	33 (7.6)	82 (19.6)	
Number of abortions				
None	361 (67.2)	324 (74.1)	272 (64.9)	< 0.001
Once	121 (22.5)	99 (22.7)	80 (19.1)	
≥ 2 times	55 (10.2)	14 (3.2)	67 (16.0)	
Number of prior live births				
0	418 (77.8)	362 (82.8)	327 (78.0)	0.334
1	109 (20.3)	68 (15.6)	83 (19.8)	
≥2	10 (1.9)	7 (1.6)	9 (2.1)	
History of pregnancy complications				
Yes	37 (6.9)	34 (7.8)	75 (17.9)	< 0.001
No	500 (93.1)	402 (92.2)	344 (82.1)	
Current pregnancy was planned				
Yes	395 (73.6)	343 (78.5)	348 (83.7)	0.001
No	142 (26.4)	94 (21.5)	68 (16.3)	
Agrees with the necessity for maternal healthcare				
Yes	460 (85.7)	405 (92.7)	359 (85.9)	0.001
No	77 (14.3)	32 (7.3)	59 (14.1)	
Agrees with the necessity for maternal healthcare when feeling good				
Yes	498 (92.7)	415 (95.0)	398 (95.0)	0.225
No	39 (7.3)	22 (5.0)	21 (5.0)	
Delivery location was predominantly selected by the woman				
Yes	422 (78.6)	373 (85.4)	359 (85.7)	0.004
No (i.e. selected by others)	115 (21.4)	64 (14.6)	60 (14.3)	
Health insurance schemes				
Yes	467 (87.0)	375 (85.8)	365 (87.1)	0.824
No	70 (13.0)	62 (14.2)	54 (12.9)	
Distance to hospital				
< 5 km	219 (40.8)	189 (43.3)	143 (34.2)	0.034
5-10 km	218 (40.6)	163 (37.4)	172 (41.2)	
> 10 km	100 (18.6)	84 (19.3)	103 (24.6)	
Mode of transport to the hospital				
Private cars	177 (33.1)	181 (41.4)	177 (42.7)	< 0.001
Public transport	172 (32.1)	119 (27.3)	144 (34.7)	
Taxi	38 (7.1)	26 (5.9)	40 (9.6)	
On foot	148 (27.7)	111 (25.4)	54 (13.0)	
Waiting time to see the doctor				
< 15 min	149 (27.7)	87 (20)	45 (10.7)	< 0.001
15–30 min	323 (60.1)	262 (60.1)	267 (63.8)	
31–60 min	55 (10.3)	77 (17.7)	88 (21.0)	
> 60 min	10 (1.9)	10 (2.2)	19 (4.5)	
Subjective feeling that the antenatal advice applies to their current situation				
Yes – it does	515 (95.9)	417 (95.4)	408 (98.1)	0.084
No – it does not	22 (4.1)	20 (4.6)	8 (1.9)	

*Note.* P is from a chi-squared test. Age take 35 as cutoff for pregnant women aged more than 35 usually be called elderly pregnant women

Multinomial logistic regression analysis with primary hospital as the dependent variable was performed to reveal the factors associated with attendance at each level of hospital (Table 2).

**Table 2:** Multinomial logistic regression analysis of factors associated with pregnant women attending different levels of hospital in Guangdong, China (*n* = 1393)

	Secondary vs primary hospital			Tertiary vs primary hospital		
	B	*P*-value	OR [95% CI]	B	*P*-value	OR [95% CI]
Overall	−1.551	0.035		1.602	0.016	
Distance to hospital, vs > 10 km						
< 5 km	−0.296	0.148	0.74 [0.50,1.11]	− 0.490	0.022	0.61 [0.40,0.93]
5–10 km	−0.419	0.032	0.66 [0.45,0.96]	−0.474	0.017	0.62 [0.42,0.92]
Waiting time to see the doctor, vs > 60 min						
< 15 min	−0.435	0.385	0.65 [0.24,1.73]	−1.731	< 0.001	0.18 [0.07,0.44]
15–30 min	0.037	0.939	1.04 [0.40,2.70]	− 0.642	0.139	0.53 [0.23,1.23]
31–60 min	0.528	0.304	1.7 [0.62,4.63]	−0.170	0.713	0.84 [0.34,2.09]
Mode of transport vs on foot						
Private car	0.024	0.900	1.02 [0.71,1.49]	0.419	0.059	1.52 [0.98,2.35]
Public transport	−0.117	0.547	0.89 [0.61,1.3]	0.735	0.001	2.09 [1.35,3.22]
Taxi	−0.037	0.904	0.96 [0.53,1.75]	0.982	0.002	2.67 [1.45,4.92]
Annual per capita household income, < vs ≥ 50,000¥	0.281	0.064	1.32 [0.98,1.78]	−0.277	0.082	0.76 [0.56,1.04]
Urban vs rural living	0.328	0.033	1.39 [1.03,1.88]	0.456	0.006	1.58 [1.14,2.18]
Planned pregnancy, yes vs no	0.072	0.659	1.08 [0.78,1.48]	0.424	0.020	1.53 [1.07,2.19]
History of complicated pregnancy, yes vs no	0.456	0.101	1.58 [0.92,2.72]	0.852	0.001	2.35 [1.43,3.86]
Necessity for maternal healthcare, yes vs no	0.549	0.019	1.73 [1.1,2.74]	−0.239	0.268	0.79 [0.52,1.20]
Previously participated in medical examination before pregnancy, yes vs no	0.187	0.190	1.21 [0.91,1.59]	0.108	0.473	1.11 [0.83,1.50]
Number of previous pregnancies, vs ≥ 3						
1	0.466	0.224	1.59 [0.75,3.38]	0.143	0.729	1.15 [0.51,2.59]
2	0.585	0.070	1.80 [0.95,3.38]	0.674	0.058	1.96 [0.98,3.94]
Number of prior abortions, vs ≥ 2						
None	0.709	0.140	2.03 [0.79,5.2]	−0.526	0.239	0.59 [0.25,1.42]
1	0.768	0.067	2.16 [0.95,4.90]	−0.939	0.014	0.39 [0.19,0.83]
Age, < 35 vs ≥ 35 years	−0.351	0.271	0.70 [0.38,1.32]	−0.975	0.001	0.38 [0.21,0.68]
Decision-making power of delivery, yes vs no	0.369	0.043	1.45 [1.01,2.07]	0.221	0.254	1.25 [0.85,1.82]
Highest education level, vs college or above						
Junior high school or below	−0.687	0.001	0.5 [0.34,0.75]	−1.171	< 0.001	0.31 [0.2,0.49]
High school	−0.779	< 0.001	0.46 [0.33,0.65]	−0.966	< 0.001	0.38 [0.27,0.55]

*Note.* *B* Estimated multinomial logistic regression coefficients for the models; *CI* Confidence interval, *OR* Odds ratio

Pregnant women who attended a primary hospital were significantly more likely to have travelled 5 to 10 km from the hospital (vs >  10 km), reside in a rural area, or have a lower education level than those attending a secondary or tertiary hospital. Pregnant women attending a primary hospital were more likely to have travelled less than 5 km, waited less than 15 min to see a doctor (vs ≥ 60 min), have an unplanned pregnancy, no history of a complicated pregnancy, had one previous abortion (vs ≥ 2), or be aged < 35 years, than those who attended a tertiary hospital.

Conversely, women who attended a secondary hospital vs a primary hospital were more likely to believe it necessary to seek maternal healthcare and were more likely to have made the decision on the location of the delivery themselves. Women attending a tertiary hospital vs a primary hospital were more likely to have travelled to the hospital by public transport or taxi (vs on foot).

## Discussion

Andersen’s health behavioral model is the cornerstone of many studies assessing patient healthcare-seeking behavior and the accessibility of health services. The model has been used to improve healthcare services worldwide [11, 12, 23]. Our results demonstrated that there were significant demographic/pregnancy and environmental factor differences among pregnant women attending primary, secondary and tertiary hospitals.

Several of the identified factors were consistent with the known risk factors for pregnancy, indicating that the national prenatal health service in China was functioning well at the time of the survey. For example, our study showed that pregnant women of advanced maternal age (AMA) (aged 35 years or older) were more likely to be attending a tertiary hospital than a primary hospital for antenatal care. AMA is widely recognized as a risk factor for pregnancy complications and adverse pregnancy outcomes [24–26]. Although we cannot determine the reasons for each hospital visit, the finding that advanced women were attending a tertiary hospital suggests that these women were receiving more specialized care.

Health Commission of Guangdong Province released that the incidence of ‘high risk pregnancies’ is increasing, and significantly so, from 12% in 2013 to 30% in 2015 [27]. At the beginning of 2016, the Chinese health and family planning commission implemented a ‘two-child policy’, which is expected to increase the number of pregnancies and births, particularly to older mothers. The current healthcare-seeking behavior findings suggest that this may put additional demand on tertiary hospitals; thus, early assessment of all pregnant women at primary or secondary hospitals should be encouraged in order to prioritize resource allocation most effectively and promoting the equity of healthcare-seeking behavior of pregnant women.

This study indicated that tertiary hospitals were more likely to be attended by pregnant women with a history of pregnancy complications, abortion, and who were having a planned pregnancy, than a primary hospital. The former two groups of pregnant women chose the tertiary hospitals mostly out of the consideration of their actual medical needs, in line with previous studies [28]. However, for those women with planned pregnancy, seeking healthcare in the tertiary hospitals probably only because of them being cautious and attentive to the needs of the current pregnancy, rather than medical needs, this may not be encouraged from the perspective of health equity. Interestingly, a New Zealand study has shown no difference of clinical outcomes of women giving birth in either a tertiary level maternity hospital or a freestanding primary level maternity unit [29]. This paper concluded that women’s experience of transfer to a primary hospital was generally positive, suggesting that patients can be reallocated between hospitals during pregnancy. For example, a patient attending a tertiary hospital could be asked to attend a primary hospital where the clinical conditions allow, freeing up specialized resources for those with the most need. For health system and standard hospital facilities are different in China, convenient reallocation are impossible in most areas currently. Thankfully, a series of policies are being carried out, which make it possible someday.

Our findings also showed women attending a secondary or tertiary hospital were more likely to reside in an urban area, or have received a higher level of education than those attending the primary hospital. These findings are similar to the earlier research of Yanikkerem et al [30]*.* Large differences in healthcare accessibility in urban and rural areas is commonly observed in many countries, for example the Philippines and Cambodia [31, 32]. In the province of Guangdong, China, the distribution of tertiary hospitals favors urban areas. Half of all tertiary hospitals are located in the city of Guangzhou, whereas most other cities in the region have one or no tertiary hospitals. According to the China Statistical Yearbook, 178 township health centers and 16 community health centers in Guangdong hadn’t delivered any babies during 2015, and 412 maternity beds were idle. This indicates that there is a large pool of unutilized resources in primary care locations. This study suggested that pregnant women with higher level of education were potential target for interrupting of healthcare seeking behavior.

Secondary hospitals are more likely to be attended by pregnant women who considere it necessary to seek maternal healthcare and who decide for themselves where to have their baby delivered. A study has previously suggested that spouses can intensely influence the maternal healthcare sought by a pregnant woman. Men typically are the primary earners and this often manifests as also having a strong influence in a wife’s medical care [33, 34]. The influence of family in a woman’s maternal healthcare is an interesting topic and deserves further study [35]. For the aspect of equity of health, assessing the health risks of pregnant women and diverting pregnant women without medical needs to primary health care institutions are main issues that need to be considered.

Our study also suggests that convenience may be an important part of women’s choice of hospital. Attendance at a tertiary vs a primary hospital was associated with women choose public transport/taxi as their mode of transport (compared with on foot). In China, these are also the main modes of transport mode in urban areas, where the tertiary hospitals are located. These factors demonstrate that patients in rural areas may find access to specialist medical attention to be more difficult, and this should be a consideration when defining future healthcare investment and policy. Long waiting times in tertiary hospitals is also an ongoing challenge for the national health service [36–38].

This research is limited in that it only analyzed selected, pre-defined, environmental and demographic/pregnancy characteristics. Other factors may also be important, but were not surveyed. As this is a cross-sectional study, the results may infected by respondent bias. The interplay between the factors in individual patients could not be assessed due to the nature of the analysis.

## Conclusion

One environmental factor (urban vs rural living), and several demographic/pregnancy characteristics (a planned pregnancy, history of a complicated pregnancy, perceived necessity for maternal healthcare, number of prior abortions, woman’s age, highest education level, distance to the hospital, waiting time, mode of transport, power to make decision about delivery location) were found to be associated with pregnant women’s choice of hospital in Guangdong, China. To optimize the efficiency of the national health service, we have an idea that women with low risk pregnancies should be initially encouraged to attend a primary or secondary hospital for antenatal care, which could relieve the pressure on resources in tertiary hospitals. Of course, there are lots of tasks such as the safety, cost and efficiency of care delivered at various sites should be studied in future researches.

## Data Availability

All the data relevant for the manuscript are reported in tables. The raw data can be accessed from the corresponding author up on request.
